# The lifecycle and evolution of new regimens on the National Comprehensive Cancer Network Guidelines for newly diagnosed multiple myeloma

**DOI:** 10.1093/oncolo/oyae332

**Published:** 2024-12-24

**Authors:** Ghulam Rehman Mohyuddin, Jehad Almasri, Aaron Goodman, Alyson Haslam, Vinay Prasad

**Affiliations:** Division of Hematology, Huntsman Cancer Institute, The University of Utah, Salt Lake City, UT 84112, United States; Division of Hematology, University of Cincinnati, Cincinnati, OH 45221, United States; Division of Hematology, University of California San Diego, La Jolla, CA 92093, United States; Division of Epidemiology and Biostatistics, University of California San Francisco, San Francisco, CA 94158, United States; Division of Epidemiology and Biostatistics, University of California San Francisco, San Francisco, CA 94158, United States

**Keywords:** myeloma, guidelines, regimens, approvals

## Abstract

**Introduction:**

Prior studies have evaluated the level of evidence behind treatment options listed in the National Comprehensive Cancer Network (NCCN) guidelines, but no study has categorized the life cycle of regimens listed in the NCCN guidelines. We longitudinally assessed the life cycle for each regimen for newly diagnosed multiple myeloma. We track the date of first clinical data, the date of regimen addition to NCCN guidelines, the date phase 3 data (if performed) were reported, and the results of phase 3 trials.

**Methods:**

We systematically examined NCCN guidelines from January 2000 to April 2021. The primary objective of our study was to assess the life cycle of each drug/regimen listed on the NCCN guidelines. We systematically examined the following aspects for each regimen: (1) the inception of prospective clinical data, (2) its inclusion in the NCCN guidelines, (3) the completion of a randomized trial (if done), (4) the presence of an overall survival benefit in such trials, and (5) the removal of a regimen from NCCN guidelines (if done) and its corresponding timeline.

**Results:**

Twenty-one regimens were added across 50 NCCN guideline document iterations during a 22-year period. The median time from when clinical data were first presented to when a regimen was first listed in the guidelines was 15 months. Phase 3 studies were conducted for 17 regimens (80%), with a surrogate endpoint (response rate or progression-free survival) as endpoint for all trials, other than one. The median time from a regimen being included in the NCCN guideline to its phase 3 data publication was 43 months. The primary endpoint was met for 13 trials (81%). No regimen was removed for a phase 3 endpoint not being met. Six regimens (38%) showed overall survival benefit. Five (23%) regimens were removed from NCCN guidelines, with none being due to failure in phase 3 testing.

**Conclusion:**

Myeloma NCCN guidelines remain relevant and current, adding new regimens with promising early-phase data, and removing regimens that become obsolete over time. However, this process is inconsistent and may benefit from standardization.

**Implications for Practice**

Myeloma regimens appear in NCCN guidelines before randomized trial results, potentially offering earlier access to beneficial therapies.

Regimens are removed from the NCCN guidelines not due to failure of confirmatory trials, but due to the availability of better alternate therapies over time.

The process of addition and removal of myeloma regimens in the NCCN guidelines remains inconsistent and may benefit from standardization.

## Introduction

In the last 2 decades, there has been tremendous progress for patients with multiple myeloma (MM), including over 15 approvals across multiple drug classes.^1^ The National Comprehensive Cancer Network (NCCN) guidelines list available treatment options for MM for all lines of therapy. In 1993, as part of the Omnibus Budget Reconciliation Act, Congress mandated that Centers for Medicare and Medicaid Services use expert compendiums to determine coverage decisions for off-label drugs used in cancer care.^2^ In 2012, NCCN became listed as a compendium, and at present, private insurance companies also base their reimbursement decisions based on NCCN guidelines. As a result, current NCCN guidelines impact coverage decisions for most patients with cancer in the USA.^3,4^ Although various studies have previously evaluated the level of evidence behind treatment options listed in NCCN guidelines,^5-7^ no study has categorized the evolution and life cycle of specific treatment options listed on the NCCN guidelines for a specific disease state.

In this study, we categorize the lifecycle of changes to NCCN guidelines for newly diagnosed MM by tracking (1) the date when any clinical data were first reported (eg, a single arm phase 1 or 2 study), (2) the date of regimen addition to the NCCN guidelines, (3) the date a randomized, phase 3 trial was reported, (4) whether that randomized trial met the primary endpoint, and (5) whether it also demonstrated an improvement in overall survival. Finally, we noted, (6) whether trials not meeting endpoints led to removal of the regimen from the guidelines. We sought to characterize changes to these guidelines over time, by ascertaining each published guideline in detail over a 22-year period, including the number of pages, the number of references, and the number of listed options and changes over time.

## Methods

As there was no direct patient care or patient information obtained during this study, this study was considered exempt from the Institutional Board Review requirement.

We obtained all NCCN guidelines for MM from January 2000 to April 2021 by requesting them from the NCCN via email. The final data analysis was done in October 2023 to allow for follow-up from the changes in 2021 and to allow analysis of any new publications that may have resulted since the guidelines were published in April 2021. The primary objective of our study was to assess the lifecycle of each drug or regimen listed on the NCCN guidelines. This was done in a sequential fashion. We systematically examined the following aspects for each regimen: (1) the inception of prospective clinical data, (2) its inclusion in the NCCN guidelines, (3) the completion of a randomized trial (if done), (4) the presence of an overall survival benefit in such trials, and (5) the removal of a regimen from NCCN guidelines (if done) following a negative confirmatory randomized trial and its corresponding timeline. We used a previously published systematic review to identify randomized trials,^8^ and a search on PubMed and Google Scholar using the terms “myeloma,” “plasma cell dyscrasia,” and the specific drugs in question was also done during June-July 2023 to identify prospective trials.

A key secondary objective of our study was to ascertain changes in the guidelines themselves over time, as the treatment options for myeloma have evolved. This would be done by ascertaining the number of NCCN guideline publications per year, the number of new changes per each published NCCN guideline, the number of references, and the number of pages per NCCN guideline and its trends over time. Two authors (G.R.M. and J.A.) independently reviewed each NCCN guideline and collected information.

Descriptive statistics were calculated. Statistical calculations were done using SPSS Version 29 (IBM). Graphs were generated using Excel and R statistical Software, version 4.2.1., package “ggplot2.” Statistical analyses were done by 2 authors (G.R.M. and A.H.).

## Results

### Characteristics of published guidelines

**Figure 1.**
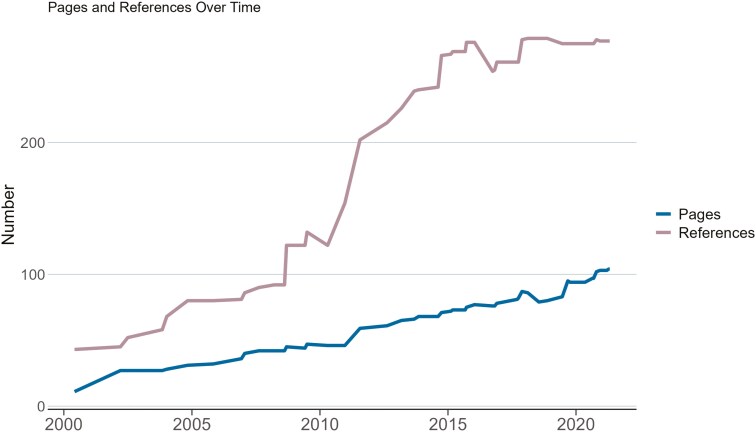
The number of pages and references for the National Comprehensive Cancer Network guidelines over time.

A total of 50 NCCN guidelines addressing MM were published from January 2000 to April 2021. The number of pages ranged from only 11 for the first guideline published in June 2000 to 104 in April 2021 (median = 72.5, Q1 = 44, Q3 = 86, IQR = 42). The number of references increased from 43 for the guideline in June 2000 to 277 in July 2021 (median = 261, Q1 = 122, Q3 = 275, IQR = 153). Figure 1 highlights the changes in the number of pages and references of the NCCN guidelines over time.

### Timing of clinical data generated

**Figure 2.**
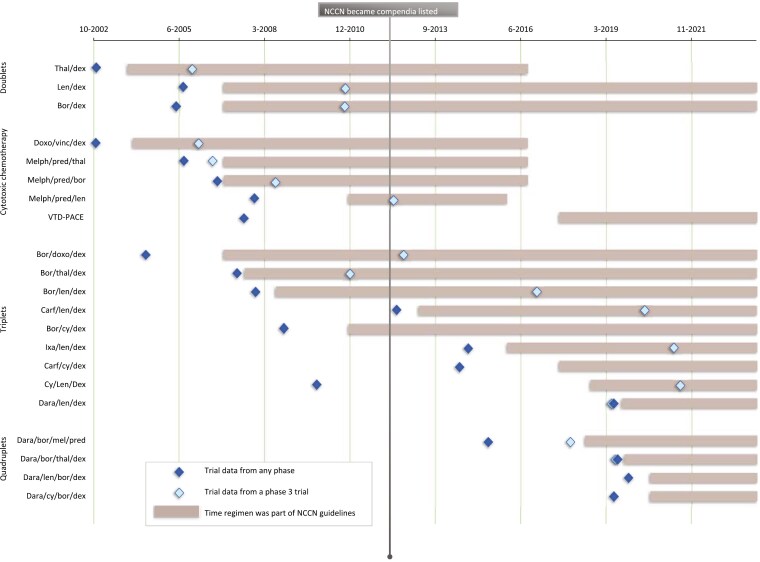
Lifecycle of myeloma regimens included in the National Comprehensive Cancer Network guidelines and the publication of trial results supporting their efficacy. Regimens are classified as follows: cytotoxic chemotherapy (other than low-dose cyclophosphamide), doublets, triplets, and quadruplets. Abbreviations: Thal: thalidomide, Dex: dexamethasone, Doxo: Doxorubicin, Vinc: Vincristine, Mel: Melphalan, Pred: Prednisone, Bor: Bortezomib, Len: Lenalidomide, Carf: Carfilzomib, Dara: Daratumumab, Ixa: Ixazomib, VTD-PACE: Bortezomib-thalidomide-dexamethasone-cisplatin-doxorubicin-cyclophosphamide-etoposide.

**Figure 3.**
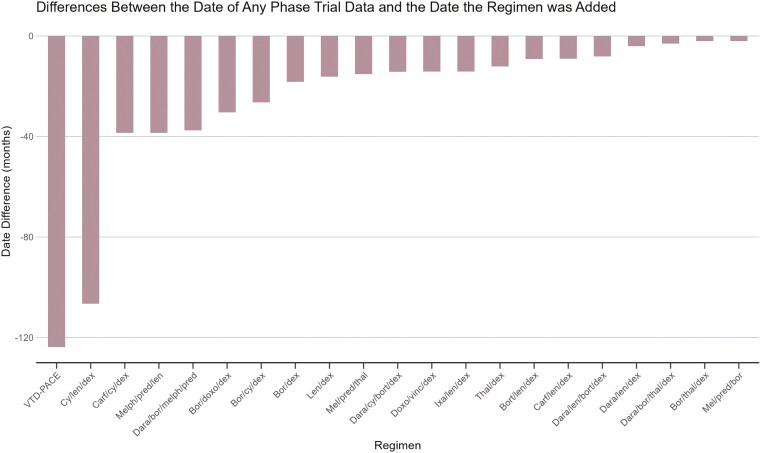
Differences (in months) between when a regimen were added to the National Comprehensive Cancer Network guidelines and when the prospective clinical data for the regimen were first published.

The lifecycle of each regimen is listed in Figure 2, organized according to classes of drugs. The median time from when clinical data were first presented or published for a regimen to when it was listed in the NCCN guidelines was 15 months (Q1 = 9, Q3 = 37, IQR = 28). This is graphically represented in Figure 3.

**Figure 4.**
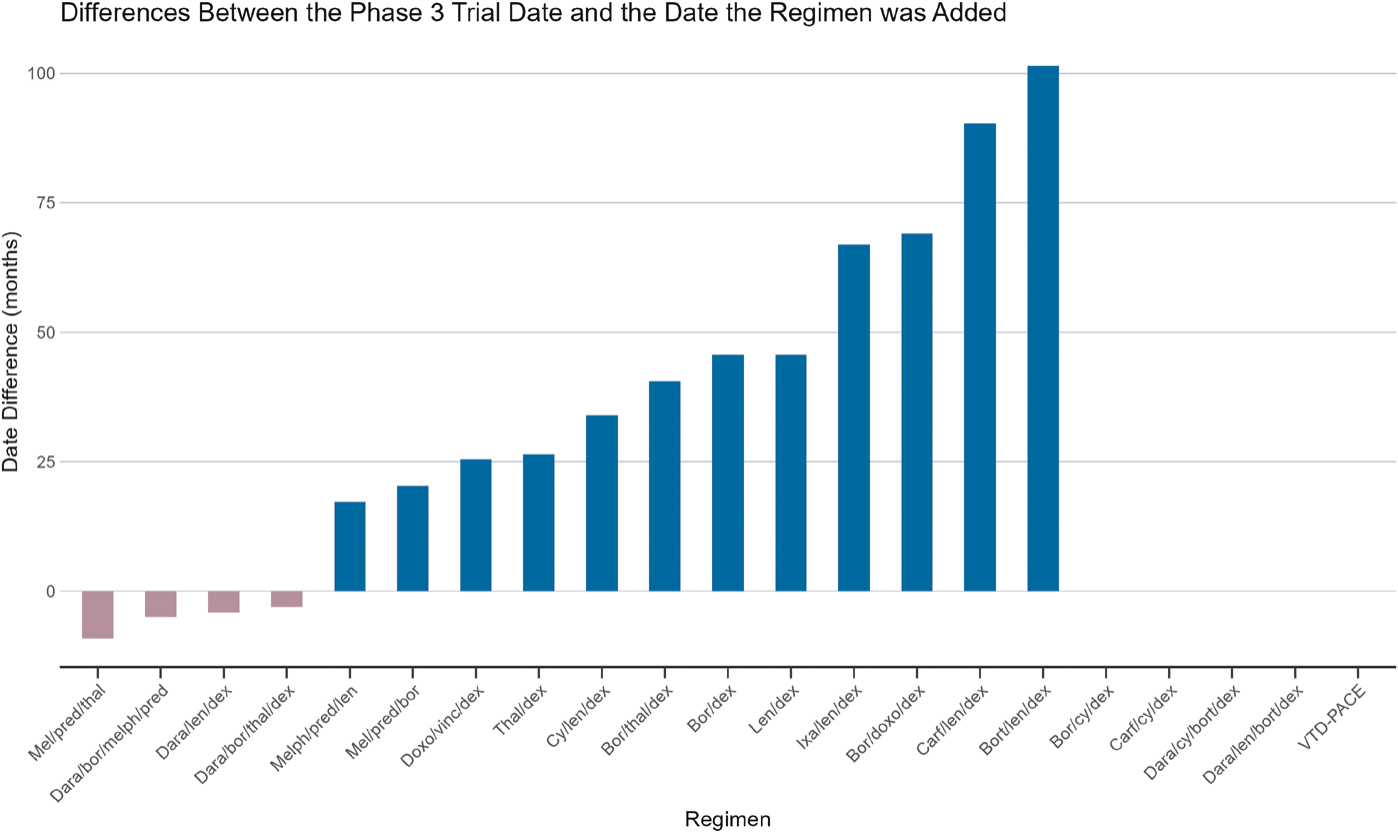
Differences (in months) between when a regimen was added to the National Comprehensive Cancer Network guidelines and when Phase 3 data were first available.

The median time from NCCN guideline listing to the publication/abstract presentation (whichever came first) of phase 3 data was 43 months (Q1 = 26, Q3 = 67, IQR = 41) for studies where phase 3 data followed NCCN guideline listing. The date of phase 3 publication ranged from as early as 9 months prior to NCCN guidelines listing (such as data from melphalan/prednisone/thalidomide^9^) to up to 102 months after being listed (for bortezomib/lenalidomide/dexamethasone^10^). This is graphically represented in Figure 4.

**Figure 5.**
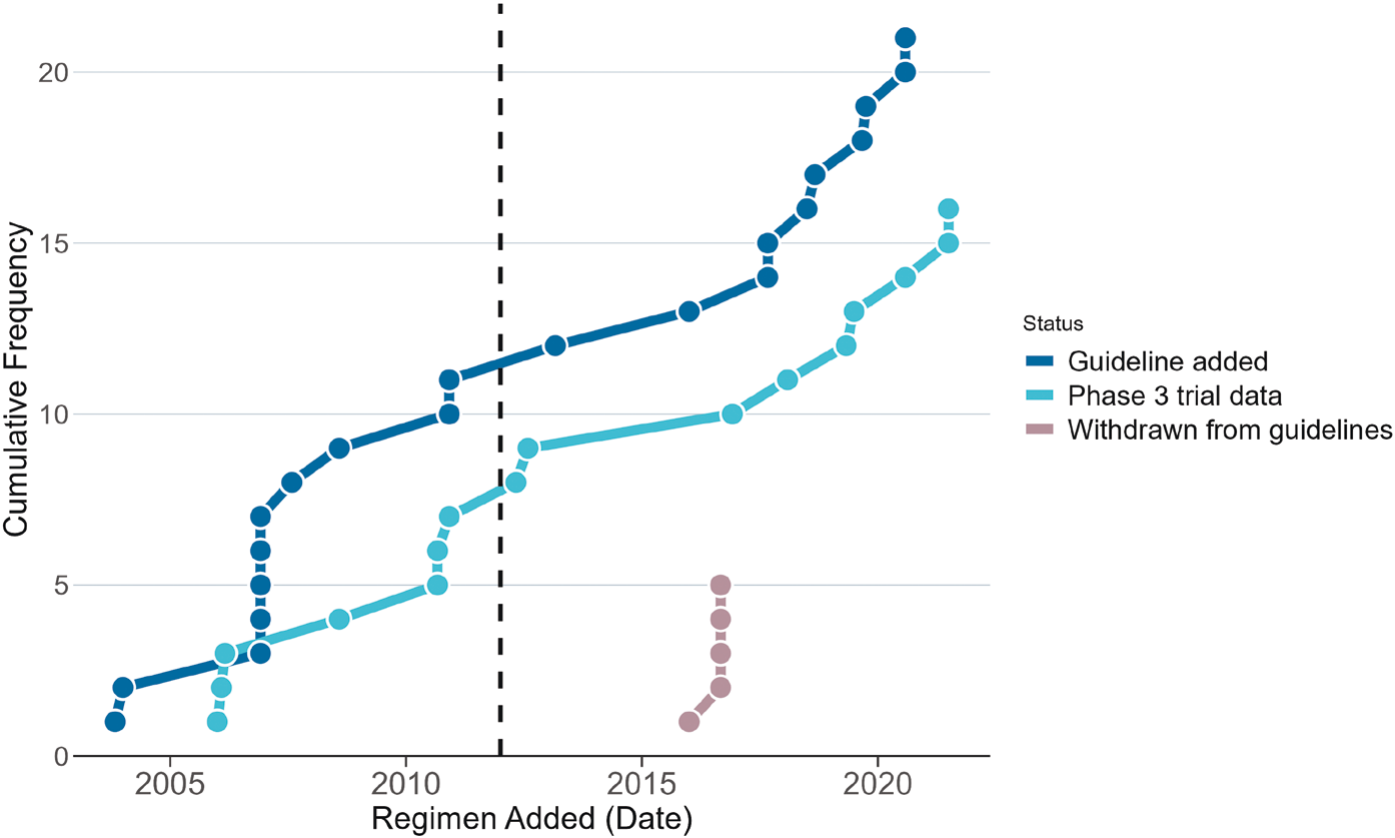
The cumulative frequency of changes (addition, phase 3 trial data, and withdrawals) in National Comprehensive Cancer Network (NCCN) myeloma regimen guidelines. The dashed line is when NCCN guidelines became compendia listed.

There were 21 new regimens/options during this timeframe. They are represented in Figure 2 according to the year they were added. Noteworthily, 5 new additions were included in 2006, and 2 each in the years 2017, 2018, 2019, and 2020. Among the 21 new regimens added during our study time-period, only 5 regimens (24%) were explicitly removed, whereas the others remained listed on the guidelines. For each of the 5 regimens that were removed, the confirmatory randomized trials for all 5 regimens had successfully met their endpoint, indicating that the removal was likely due to other reasons (such as availability of other agents with perceived better safety and efficacy). Figure 5 is a cumulative frequency figure that graphically represents changes to the NCCN guidelines (addition, publication of phase 3 data, and withdrawals) during our study time.

### Generation and timing of phase 3 data with respect to NCCN guideline addition

Among the 21 new regimens added, confirmatory phase 3 studies were performed for 17 of these regimens (80%). For 4 regimens, no phase 3 trial had been performed at the time of analysis. These regimens include bortezomib, cyclophosphamide, and dexamethasone (added December 2010); bortezomib, thalidomide, and dexamethasone with the addition of multiagent cytotoxic chemotherapy (VTD-PACE, added September 2017); carfilzomib, cyclophosphamide, and dexamethasone (added September 2017, although a phase 2 trial [FORTE] did evaluate this regimen); and daratumumab, cyclophosphamide, bortezomib, and dexamethasone (added August 2020).

For 4 regimens, phase 3 data preceded the regimen’s addition to the NCCN guidelines, namely melphalan, prednisone, thalidomide^9^ (9 months prior); daratumumab, bortezomib, melphalan, prednisone^11^ (5 months prior); daratumumab, lenalidomide, dexamethasone^12^ (4 months prior); and daratumumab, bortezomib, thalidomide, dexamethasone^13^ (3 months prior). For the remaining 12 regimens where entry into NCCN guidelines preceded phase 3 data, it took a median of 45 months (Q1 = 25, Q3 = 68, IQR = 43) for phase 3 data to become available.

For the 17 regimens for which a phase 3 trial was conducted, 15 of these trials met their predetermined endpoint (88%), and 2 did not meet the endpoint (12%).

Both regimens that did not meet their endpoint (carfilzomib/lenalidomide/dexamethasone^14^ and ixazomib/lenalidomide/dexamethasone^15^) continued to be listed in the most recent guidelines, although the trial evaluating carfilzomib/lenalidomide/dexamethasone in the newly diagnosed setting (ENDURANCE) was not considered to be a regulatory trial.^14^ This trial was a superiority trial, not a noninferiority one. Although both regimens may be considered to have similar activity by clinicians, it was considered to have not met its endpoint in this analysis.

### Endpoints ascertained and demonstration of overall survival benefit

For the 17 situations where a phase 3 trial was conducted, the primary endpoint was a surrogate endpoint in 16 of these trials, namely progression-free survival (or event-free survival) in 12 cases (75%) and response rate in 4 cases (25%). In one trial, the primary endpoint was a composite endpoint of PFS or OS.^16^

An overall survival benefit was shown either in original publication or follow-up publication for 7 of these 17 trials (41%) namely: bortezomib/doxorubicin/dexamethasone,^17^ melphalan/prednisone/bortezomib,^18^ bortezomib/thalidomide/dexamethasone,^19^ bortezomib/lenalidomide/dexamethasone,^10^ daratumumab/bortezomib/melphalan/prednisone,^20^ cyclophosphamide/lenalidomide/dexamethasone,^16^ and daratumumab/lenalidomide/dexamethasone.^21^ For 4 of these 7 regimens, a survival benefit was seen at the time of initial phase 3 results publication,^10,16-18^ whereas for the remaining 3, it was seen in a subsequent publication.^19-21^

## Discussion

In this first-ever analysis of the lifecycle of regimens listed on the NCCN guidelines for a cancer, we demonstrate 5 key findings. First, the NCCN has steadily expanded the number of options in newly diagnosed myeloma. We note that 21 regimens were added over the last 22 years, and only 5 were removed—a net gain of 16 regimens. This expansion reflects the growing array of therapeutic options available for treating myeloma. This is also reflected in the markedly expanding options, reference, and page count in each guideline document, which makes choosing the right regimen for a patient increasingly difficult.

Second, we find a median delay of 15 months between the first appearance of clinical trial data and addition to NCCN guidelines, with wide variability (from only 2 months for melphalan/prednisone/bortezomib to over 10 years for VTD-PACE). It is unclear why some regimens took so long to be added to the guidelines. While confirmatory trials are being conducted, regimens with promising data should be promptly added to the guidelines, allowing for easier access to therapies, although this is more relevant for relapsed/refractory myeloma where there may be less options available, rather than newly diagnosed myeloma.

Third, drugs/regimens listed on the NCCN guidelines precede a randomized trial reporting by a median of 45 months, and these randomized trials almost always utilize a surrogate outcome such as progression-free survival as a primary endpoint rather than overall survival. It is likely that listing these options before randomized trials allows for access to regimens that ultimately redefine the standard of care and improve outcomes. Given that measurable residual disease is now an approved endpoint for trials, this timeframe may be shortened even further in the future, as there will be quicker read-outs for trials.^22^

Fourth, we find that for some regimens, randomized trials were never performed and that some regimens have not yet shown a survival benefit in a randomized trial. Consider 2 examples: the triplet bortezomib/lenalidomide/dexamethasone first had clinical data in the newly diagnosed setting in November 2007^23^ and became listed on the NCCN guidelines in August 2008. The confirmatory randomized trial was not reported until December 2016,^10^ which showed an overall survival advantage 9 years from when clinical data were first presented on this regimen in the newly diagnosed setting and 8 years after it was added to the guidelines. In this instance, allowing this regimen to be listed on NCCN guidelines well in advance of randomized data, provided access to a regimen that eventually turned out to be beneficial to patients. We acknowledge that the lack of a demonstrated survival benefit to date does not mean that these regimens are ineffective or that there is no potential survival benefit. These trials were not necessarily powered to detect survival benefits, and there may not have been sufficient power or follow-up to ascertain this. Alternatively, in cases where survival benefit was seen, that may not be recapitulated in the US marketplace where access to good therapies exists upon progression, as postprogression therapies in global myeloma studies are often not fully reported or up to the US standard.^24^ Furthermore, this has also led to options being listed (and continuing to be listed) despite failing confirmatory randomized trials years later, such as the triplet ixazomib/lenalidomide/dexamethasone.^15^

Finally, we find that 5 regimens were ultimately removed from the guidelines. We show that the removal of regimens was not due to adverse clinical trial data, or failure to achieve a response but rather due to the availability of other newer agents. Among the 5 regimens removed, 4 were alkylator-based regimens (doxorubicin/vincristine, dexamethasone, melphalan/prednisone/thalidomide, melphalan/prednisone/bortezomib, and melphalan/prednisone/lenalidomide), with the fifth being thalidomide/dexamethasone. We hypothesize that these regimens were removed due to the availability of agents thought to be either safer or more active than these regimens. Of note, the doublet of lenalidomide/dexamethasone showed a progression-free survival and overall survival advantage compared with melphalan/prednisone/thalidomide,^25^ likely influencing the decision to keep lenalidomide/dexamethasone backbone regimens listed on these guidelines while removing others.

Our findings highlight that NCCN guidelines for newly diagnosed myeloma have generally remained relevant and current, shedding regimens that have become obsolete over time. However, this process could benefit from standardization.

Our recommendations to standardize and improve this process are as follows. If the goal of the NCCN guidelines is to provide a laundry list of options that can be used and reimbursed by payers, then all regimens with promising early-phase data should be promptly added within 1-2 months of data availability, and the current variability in adding these regimens stands to be improved. Regimens that are added based on early phase data should point to a future confirmatory trial that is being conducted and have a clear timeline upon which such a regimen should be re-evaluated based on the future confirmatory trial. The magnitude of benefit in a surrogate outcome for such a future randomized trial should be clearly defined apriori for the regimen to continue to be listed. This is particularly relevant given that MRD is now acceptable as an acceptable primary endpoint.^22^ As an example, the recent trial of isatuximab, carfilzomib, lenalidomide, dexamethasone versus carfilzomib, lenalidomide, and dexamethasone showed a difference in the primary endpoint of MRD negativity of 10% (77% achieving MRD negativity at the 10^−5^ sensitivity threshold in 4 drug arm vs 67% in the 3 drug arm, *P* =.049).^26^ Whether a delta of 10% in this surrogate outcome is sufficient to be listed on NCCN guidelines is an important question for the field to grapple with and such thresholds should be defined apriori, given the importance of the NCCN in reimbursement and decision-making. Furthermore, if a randomized trial fails to demonstrate a clear benefit, such as ixazomib, lenalidomide, and dexamethasone in its confirmatory trial, such a regimen should be removed. We acknowledge that removing regimens that have become obsolete over time is difficult to standardize, and that the current process has served well to remove older regimens.

Although our work is limited to newly diagnosed MM, this methodology can be applied to other cancers to track the longitudinal life cycle of a particular regimen. Such a model can be used to track whether the guidelines are consistently and promptly adding regimens based on promising early phase data and whether regimens are removed due to failure of confirmatory trials or whether the regimens have become obsolete over time. A close evaluation of such processes can serve to improve the quality and applicability of guidelines in the future.

Our work has limitations. First, it is limited to newly diagnosed myeloma, and it does not reflect changes in the relapsed/refractory space, where new drugs are often studied and approved first. Showing an overall survival difference in this space may take a very long time, and the inability to have shown such a difference to date may not be reflected adversely on the regimen. Second, we did not analyze the grading of evidence behind each recommendation, although previously published work has already analyzed this.^5-7^ Finally, although a phase 3 trial of cyclophosphamide/bortezomib/dexamethasone technically exists,^27^ this utilized such high dosages of cyclophosphamide compared with currently used dosages of cyclophosphamide in combination regimens, that we did not consider this to be a representative trial of cyclophosphamide/bortezomib/dexamethasone in its currently used format. We acknowledge that although the findings of the study are US centric, the US drug approval and utilization has profound implications on both subsequent approvals and practice across the world.

In summary, we demonstrate that the NCCN guidelines for myeloma have significantly expanded in scope, length, and complexity over time. Drugs and regimens are often added to the guidelines more than a year after initial trial reports but several years before randomized data results are presented, allowing for earlier access to these drugs. For some listed regimens, no randomized data have ever validated their inclusion. While some changes and additions facilitate earlier access to therapies that ultimately alter the disease’s natural history (such as bortezomib/lenalidomide/dexamethasone), other unproven or potentially ineffective therapies also remain listed. Although the prompt addition of new, potentially effective regimens undeniably provides early access to drugs, the processes of adding and removing regimens from the guidelines remain inconsistent and subjective and may benefit from standardization.

## Data Availability

All data in this manuscript were gathered from NCCN guidelines which are available upon request from the NCCN. Our dataset can be shared upon request to the corresponding author.
